# American Dental Association

**Published:** 1885-09

**Authors:** 


### Reports of Society Meetings.

### AMERICAN DENTAL ASSOCIATION.

### TWENTY-FIFTH ANNUAL MEETING, HELD AT MINNEAPOLIS, MINN., AUGUST 4, 5, 6, AND 7, 1885.

REPORTED FOR THE "INDEPENDENT PRACTITIONER," BY "MRS. M. W. J."

The twenty-fifth annual meeting of the American Dental Association was held at Minneapolis, commencing Aug. 4, 1885. This was in many respects the most successful meeting ever held. The attendance was unusually large, the number of new members admitted (103) unprecedented; the papers read were marked by deep and thorough scientific research and investigation, and the discussions were earnest and animated.

Delegates were present from all the most prominent State, District, and Local Societies of the United States, from nearly all the Dental Colleges, and the Dental Departments of several Universities. From New England to New Orleans, from the District of Columbia to the far West, north and south, east and west, all were alike impartially represented.

The officers, committees, and sections were as follows :

*President*—J. N. Crouse, Chicago, Ill.

*First Vice-President*—M. W. Foster, Baltimore, Md.

*Second Vice-President*—C. F. Rich, Saratoga, N. Y.

*Recording Secretary*—Geo. H. Cushing, Chicago, Ill.

*Corresponding Secretary*—A. W. Harlan, Chicago, Ill.

*Treasurer*—Geo. W. Keely, Oxford, Ohio.

EXECUTIVE COMMITTEE.

C. N. Pierce, *Chairman.* A. M. Dudley, *Secretary.*

FIRST DIVISION—COMMITTEE OF ARRANGEMENTS.

J. N. Crouse, Geo. L. Field, W. N. Morrison.

SECOND DIVISION—ON CREDENTIALS, ETHICS AND AUDITING ACCOUNTS.

A. M. Dudley, S. G. Perry, A. O. Hunt.

THIRD DIVISION—ON VOLUNTARY ESSAYS.

C. N. Pierce, L. D. Shepard, Geo. J. Friedrichs.

LOCAL COMMITTEE OF ARRANGEMENTS.

A. T. Smith, of Minneapolis ; F. H. Gardiner, of Chicago ; and H. J. McKellops, of St. Louis.

SECTIONS.

Section I—Prosthetic Dentistry, Chemistry, and Metallurgy. John Allen, Chairman; W. H. Trueman, Secretary.

Section II—Dental Education. A. O. Hunt, Chairman.

Section III—Dental Literature and Nomenclature. J. Taft, Chairman.

Section IV—Operative Dentistry. L. D. Shepard, Chairman ; V. H. Jackson, Secretary.

Section V—Anatomy, Histology and Microscopy. C. F. W. Bodecker, Chairman; Frank Abbott, Secretary.

Section VI—Pathology, Therapeutics, and Materia Medica. A. W. Harlan, Chairman; F. M. Odell, Secretary.

Section VII—Physiology and Etiology. H. A. Smith, Chairman; N. W. Kingsley, Secretary.

The meeting was called to order at 10 A. M., with the President in the chair. The first morning session was devoted to the work of organization, the usual routine business, reports of committees, etc.

Dr. E. Parmly Brown, chairman, asked that the committee appointed at the last meeting for securing larger attendance of lapsed members, be discharged, further efforts being rendered unnecessary by the present large attendance. Information was given that old members, who had lost their membership by non-attendance and non-payment of dues, would be reinstated on payment of two years' past dues.

On motion, the committee was discharged.

A special committee, consisting of Drs. Smith, of Minneapolis, Gardiner, of Chicago, and Field, of Detroit, was appointed to make arrangements for the half-day devoted to clinics.

The Committee on Publication announced that, through arrangements renewed with the S. S. White Dental Manufacturing Co., their work had been done almost without cost to the Association; that the thanks of the committee were tendered to Mr. Hise for valuable assistance rendered; that the business minutes of the Association, from its first organization in 1856, had been compiled from the original MSS., and published for the benefit of members of the Association. On motion of Dr. H. A. Smith, of Cincinnati, the transactions were ordered bound in a more substantial manner, suitable for library purposes.

The Secretary, Dr. Geo. H. Cushing, announced the death, during the past year, of Dr. J. G. Ambler, of New York, and Dr. Isaiah Forbes, of St. Louis. Drs. Taft, Spalding (of St. Louis) and Atkinson were appointed a Committee on Necrology.

Dr. T. T. Moore, of S. C., gave notice of a motion to amend Art. IV of the Constitution, by striking out "1st Tuesday" (in August) and substituting "4th Tuesday," as the time for the annual meeting. This lays over till next year for discussion and final action.

Considerable time was consumed in calling the roll of the sections and enrolling the new members by sections, sixty-three having joined the Association at the first morning session.

Adjourned to 8 P. M.

### TUESDAY EVENING SESSION.

The Committee on Voluntary Essays announced three papers, which they recommended should be read.

One from Dr. W. H. Atkinson, of New York, on "Pyorrhœa Alveolaris"; one from Dr. J. A. Robinson, Jackson, Mich., entitled

"The Painless Operation"; and one from Dr. L. C. Ingersoll, of Keokuk, Iowa, on "The Alveolar Dental Membrane." Also a paper by Dr. E. Parmly Brown, entitled "Dentistry—Its Past, Its Encouraging Present, Its Brilliant Future," which was left over from last year because of lack of time for reading.

On motion, the report was accepted.

The Treasurer reported as follows:

| | |
|---|---|
| Balance on hand, . . . . . | $1,823.95 |
| Received since last meeting, . . | 400.00 |
| | $2,223.95 |
| Expenditures as per vouchers, . | 275.00 |
| Balance on hand, . . . . . | $1,948.95 |

The report was referred to the Auditing Committee.

The President stated that his time had been so fully occupied by his duties as chairman of the Committee of Arrangements, that he had not been able to prepare the customary formal address, but he hoped the results of his labors would prove more acceptable than a speech. He would only ask the members to bear in mind that they did not come merely to elect officers and have a good time generally, but that they had legitimate work to do. He would make two recommendations: First, that more work be done by the various sections, between the annual meetings, thus facilitating work during the session; and, second, that the Association defray all expenses of legitimate scientific work and original investigation in the sections. He thought this would be the most appropriate disposition of the annual income.

This proposition was received with hearty approbation, and a committee appointed to consider the subject.

Section VII. Physiology and Etiology, reported through Dr. A. H. Thompson, Topeka, Kansas, Secretary *pro tem.*, a paper by Dr. W. C. Barrett, Buffalo, N. Y., entitled "The Earthy Phosphates." Also a recommendation that the Association appropriate the sum of $200.00 for the prosecution of scientific investigation by Section VII. Upon being called Dr. Barrett read his paper.

(A full abstract will be found on page 453. The complete manuscript, according to the terms of the contract by which the proceedings are published by the S. S. White Dental Mfg. Co., is the property of *The Dental Cosmos.*)

*Dr. C. W. Spalding,* (St. Louis)—Said that he would not attempt to refute the general position taken in the paper, but that there were some points difficult to reconcile. As a general rule the inorganic elements are not appropriated directly, as food, but, having passed through the vegetable tissues, are used advantageously. As medicine they certainly have their value. There is one inorganic substance that is largely and generally used, that would seem to constitute an exception to the rule, and that is sodium-chloride, or common salt, which is a mineral substance that is used habitually by both man and beast. It is true that it is eliminated unaltered, but it has never been shown that *all* that is taken is thus eliminated; the quantity excluded has never been proved to be equal to the quantity that is taken in. May not a portion of it undergo decomposition within the system, and the elements be incorporated into the tissues? He said that he did not assert this to be the case, but would raise the question. Wild animals seek for salt so habitually that hunters know where to look for them. With domestic animals this might be a perverted taste—a habit acquired in domesticity—but this could not be the case with the wild animals. It must be a necessity of the animal economy. Is this salt decomposed and incorporated, or is it only used in a remedial sense?

*Dr. C. N. Pierce,* (Philadelphia)—The paper sets forth ideas seemingly antagonistic to the laws governing nutrition. The idea is thrown out that man is the only animal requiring therapeutic medications or remedial agents. Now we know that some animals eat clay; the dog and the cat, which are carnivorous, eat grass, in certain conditions, as a remedy. It was said that the animal system was not competent to appropriate inorganic material, but everyone knows that if chickens are deprived of lime their eggs will have no shells, indicating some deficiency in the system. We mystify ourselves by talking of organic and inorganic elements. When lime has been taken up by vegetation it is still lime, and no less inorganic. The inorganic salts are taken indirectly through the vegetable kingdom, but they are none the less inorganic because of passing through the vegetable organism. Lime is used up by vegetation and the soil exhausted; more lime must be given to the soil to supply future crops. In certain cases we can benefit patients and aid assimilation by the administration of lime-salts. Stomach fermentation is relieved by lime water; it thus aids nutrition. Diet makes the animal; the food-habit gives form and character to the organism. Take an animal of any group—carnivorous, graminivorous, herbivorous—change the diet, accustom it to a new diet, and the whole system is changed. The different tooth-forms are the result of different diets. There is lime on the earth, in the air, in the water. Similar food-habit has made similar tooth structure; the same teeth because of the same food-habit. Dogs are carnivorous; their teeth are what they are because of their food-habit. The Polar bear is truly carnivorous; the bear of the mountains has a grinding molar because of different diet. Food and function must go hand in hand. In the human family there is a large class of children who always have a cup of fluid at hand to wash down each mouthful of food. Their teeth are soft, and soon break down; the tooth function has been delegated to the stomach. Their food contained lime enough, but the teeth were not exercised; hence the universal pathological condition called caries.

*Dr. W. H. Atkinson,* (New York)—Said he wished to congratulate Dr. Barrett on his growth; he was formerly the man *par excellence* to uphold the theory he now cries down. As he said, it is truly an easy thing to find testimony in favor of our theories, and to pass by all to the contrary. "One man's meat may be another man's poison." The same article may be food, medicine or poison, according to conditions, acting by reason of awakening latent energies roused by deficiencies in the system. The classification of organic and inorganic is simply a bedevilment of words. As the latest upheavals of mountains are the highest, so with words. We must modify our views, tear down false gods; be sure we are right, then go ahead. The subject is very complicated. We must have the ability to comprehend how pabulum is produced, or we may hammer away in vain till the crack of doom. There must be correct, serial, orderly arrangement of statement. A multiplicity of assumption is held of equal value with facts. What is the nature of the demand we call hunger? It is difficult to comprehend. It is an awakening of latent energies. We are not hungry in the stomach, but where the pabulum has been burned out. Dr. Spalding said that salt passes out without being changed; he had better revise his animal chemistry. All the oxygen which has passed within the grasp of the lungs is as dead as a door-nail, although only four per cent. has been appropriated, and it is the same with salt. Of some things we can simply say "we don't know." Dr. Pierce says the food-habit forms the teeth; that the food-habit is the mother of tooth-form, or rather of the ghost of a tooth. It is assumed that matter does all things. Scientists—so-called materialists—are the wisest in assumptions. An assumption is made as the basis of a statement. An opposite assumption cancels the first, and so on. The awakening of latent energies in so-called inorganic matters renders them capable of acting. Physicists are not willing to admit that there is any power in the atom.

*Dr. C. W. Spalding*—Does Dr. Atkinson say that salt does not pass through the system unchanged?

*Dr. Atkinson*—There must be a change in the salt, as there is in the oxygen. Oxygen that has passed through the lungs is *nil* for human inhalation, though only four per cent. has been appropriated. Salt has to be similarly changed, though not to the same extent, with oxygen.

*Dr. Spalding*—Shall we report you as saying that salt does not reappear unchanged?

*Dr. Atkinson*—Report what you please.

*Dr. Frank Abbott*, (New York)—Said he had gone through the experiments with lime phosphates, always with the same results. In no particular instance had there been marked improvement. All but one case had failed. He had obtained no better results with treatment than without. Because in certain experiments with rabbits, two having broken legs, the one treated with lacto-phosphate of lime healed more readily than the one not so treated, lacto-phosphate was given to the world as the much-needed remedy for the teeth. It succeeded with the rabbit's legs, but fails with the human teeth. He has not given up all hope, but is yet looking for the right thing. The form in which it will be assimilated has not been found; perhaps because the phosphate we buy is not pure phosphate; it is dissolved in lactic-acid; lime three parts, phosphorus one, is the ordinary formula. Except in the form of food we fail, but every person takes in his ordinary food sufficient lime-phosphate for every purpose. Ladies in certain circumstances, however, do not retain enough food; so much is rejected, from constant nausea, that they do not obtain from their food the required quantity of these elements. This is the cause of the trouble with their teeth. One speaker said that certain elements were taken to stimulate latent energies that had previously been depressed. We see the teeth and know they are defective; probably other organs are also defective, but we cannot see them as we do the teeth; hence, in the latter organs only, we recognize the deficiencies.

*Dr. Pierce*—I said the food-habit was the mother of tooth-forms. If we go into a museum with a pair of dividers and examine the lower jaw of any animal, placing one arm of the dividers on the condyles and describing the valleys in the teeth, in another class of animals placing the arm half-way between the condyles, we will find that the movement of the jaw has been the mother of the shapes of the crowns of the teeth, through the movements of the jaw.

One class—the carnivora—has dentine in the centre, enamel on the crown, and cementum on the roots ; another class has the enamel on the labial surface and cementum on the palatine surface, and the incisors always sharp. The jaw of the rodent has a lateral motion ; the enamel is not on the crown, but side by side with the dentine; the movement of the jaw has given the tooth-form.

*Dr. L. C. Ingersoll*, (Keokuk, Iowa)—Asked Dr. Pierce if he meant to say that tooth-form and function were governed by food-habit.

*Dr. Pierce*—The necessity for anything precedes its presence. In the absence of function there is absence of nutrition. Function causes nutrition ; if nutrition is delegated to another part, function follows it.

*Dr. Ingersoll*—Considered it a question whether the formation of the stomach is the result of the formation of the teeth. Is cud-chewing the mother of the double stomach ?

*Dr. Pierce*—There is much obscurity in many forms of development, but I still adhere to the principle that function is the mother of organization. The food-habit modifies the alimentary canal, as is seen by the comparative anatomy of the herbivorous and the carnivorous animals.

*Dr. C. W. Spalding*—If the food-habit controls the conformation and measurement of the jaws, I would ask when the habit originated? What governed the conformation of the first animal? If the development of stomach and teeth is contemporaneous—if the stomach only develops when the teeth indicate the need for solid food—how about the stomach when the teeth fail to appear?

If a child two years old has erupted no teeth, has it no stomach? There is a man in Pennsylvania who is forty years old, who has never had any teeth; he is in vigorous health, though he has no sweat-glands, and has no hair. Has he no stomach?

*Dr. Sitherwood,* (Bloomington, Ia.)—The results of my reading and observation agree with the position taken in the paper. There are seeming exceptions, but the rule holds good. Too much confidence is placed in feeding mineral substances. I have always been the friend of children. I left at home a sick child ten months old. Our physician has had twenty-five years more experience than I have, but when I left home I gave orders that the child should have no more of the patent "infant's foods," no mineral elements, even as medicine; only pure cow's milk and vegetable stimulants. Since my arrival I have had two dispatches, and the child is improving rapidly. The necessity of lime for egg-shells, and for salt in the human system, form two apparent exceptions to the rule, but we do not fully understand these things yet. I have no faith in administering mineral substances.

*Dr. King,* (Lincoln, Neb.)—We are assuming some things that we cannot fully sustain. Dr. Atkinson assumes that when we are hungry, it is not in the stomach, but in the part that needs to be supplied. My experience is that as soon as the stomach is filled, hunger is satisfied, though the parts that need the supplies are not yet reached. Marked results from the administration of the lime-salts cannot be expected immediately.

*Dr. Atkinson*—When what I say is impugned, I want either to prove it or be corrected. The proof that hunger is not in the stomach is seen in the common practice of the Indian, who, when he is hungry, and food is not at hand, will, by tightening his belt, bring the walls of the stomach in closer contact, so as to transmit the blood that contains food elements, and the want is satisfied, though the stomach is not filled.

*Dr. Barrett* (closing the discussion)—I wish that the subject presented in the paper had been more closely adhered to. We come here, not to discuss elementary truisms; we should be prepared to comprehend fundamental, basal principles. The hunger of the tissues is felt in the stomach. Supply the stomach with pabulum, and hunger is satisfied, but it is also satisfied with enemas. Thirst is satisfied by fluids in the stomach, but it is also satisfied just as effectively by injecting the fluid into the veins. Certain tissues can be fed without the intervention of the stomach.

Our nomenclature is deficient. The terms organic and inorganic are unsatisfactory. Water is inorganic. Because of paucity of words, our nomenclature and terminology are very unsatisfactory, but we must use common terms in common discourse. It is the general law (which has its exceptions) that the animal economy, under no circumstances, can use, for trophic conditions, inorganic substances. The vegetable organizes the inorganic, then the animal can take it. One speaker cited, as triumphant refutation, that lime was necessary for egg-shells. The shell is no portion of the egg *per se;* it simply affords extraneous protection. The lime forms this covering, but it is not essential to the egg. Egg is egg—ovum is ovum—whether in or out of the body, with or without a crystalline shell. When incubation takes place outside the body, lime is required for external protection, but it forms no part of the egg itself.

*Dr. G. J. Friedrichs,* (New Orleans)—May I ask one question?

*Dr. Barrett*—I do not like to have my argument interrupted, but go on, if the question is pertinent to the discussion.

*Dr. Friedrichs*—You stated that the shell was no part of the egg. Now it is stated in works on physiology that the young absorb lime from the shell to form the bones; therefore the shell is essential.

*Dr. Barrett*—The question is not worth the answering. Animals which are oviparous have eggs with shells. When incubation is within the body there is no shell; therefore the shell is not essential to the ovum. Inorganic elements take no direct part in nutrition; they are not built up into tissues, except as crystalline deposits in an organized matrix. Their principal function is to hold in proper solution some other essential elements. Chloride of sodium is essential in osmosis; it changes the density of certain fluids so that osmosis and exosmosis can be carried on. That is why the presence of chloride of sodium is essential. I like a sharp discussion. From flint and steel we get fire, but we cannot strike a spark from a roll of butter. From the concussion of positive minds we may strike the fire of truth.

In the carnivora the average length of the alimentary canal is five times the length of the body; in the graminivora it is thirty times. This teaches that the higher the organization of pabulum the shorter the digestive tract, or, as a general law, food elements that have been twice organized require a shorter digestive tract. For a long time we taught the administration of earthy phosphates to the mother for the nutrition of the child. This was through a misconception of the process of digestion and assimilation. The system will not take these elements from any ready-made source ; nature abhors a ready-made garment. If the tissues need the carbonate or phosphate of lime, they will be elaborated within the system, but the system will not take the inorganic carbonate or phosphate of lime and build it up into the tissues.

*Dr. Pierce,* (by consent)—It is well known that the rocks which form the foundation of the city of Paris are made up of infusorial animals. The lime was taken out of the sea by these infusoria ; these rocks are miles in extent and thousands of feet deep. This lime is already organized, having been taken up and assimilated by these microscopic animals.

*Dr. Barrett*—Do you call the product of the coral polyp organized ?

*Dr. Pierce*—It is as much organized as the lime that is taken up in grain.

*Dr. Barrett*—Vegetation does take it up and organize it; animals do not.

On motion, the Society adjourned to 9 A. M., Wednesday.

WEDNESDAY, AUG. 5—MORNING SESSION.

The minutes of the preceding session were read and approved.

Prof. Pierce, of Philadelphia, moved that a committee of three be appointed to take into consideration the suggestions of the President relative to appropriations from the treasury for the advancement of scientific investigations, and to report on the propriety, advisability, feasibility, and expediency of adopting the suggestions.

The chair appointed Dr. Pierce, of Philadelphia; Dr. A. H. Thompson, of Topeka; and Dr. E. D. Swain, of Chicago.

The discussion under Section VII was continued.

*Dr. W. H. Morgan,* (Nashville, Tenn.)—Said that one speaker had assumed that the peculiar character of development was due to food-habit. The fact was, that development followed type, the type being the result of force previously exercised. He had got the cart before the horse. Type-forms were modified by food-habit, but were not created by it. The type was also modified by exercise and use. Whenever a want is expressed in the animal economy, a desire, mental or physical, it caries with it a promise of satisfaction. There is a call for, or a need of, the salts of potassium, or of calcium, and nature has provided the supply everywhere, but he deprecated the idea of introducing the crude inorganic materials as nutrition. The line was not very clear between organic and inorganic. A potentiality was conveyed to mineral matter after it was taken into the system The human blood always has iron in it. We may administer the chemical salts of iron and increase the quantity in the system, but we do not know how much vitality it gains after the introduction. He was not prepared to take the broad ground that these elements were never incorporated into the system. There is inorganic matter in all food, and no necessity exists for introducing the crude form. When the teeth are inferior, the very material necessary to improve them is being thrown off by the system, and incrusted on the teeth. The system has a larger supply than it can appropriate. Ten times, or twenty times, as much as is needed is eliminated by the system as superfluous. This is true of the perfectly healthy body. The great trouble is in assimilation. It is digested, but not appropriated. When the matter of assimilation is regulated there will be no question of administering lime-salts.

*Dr. J. J. R. Patrick*, (Belleville, Ills.)—Said it had long been recognized as an established point that all matter was included in the two great divisions, organic and inorganic, and that there were three great kingdoms—the mineral, vegetable, and animal—and that was the order of creation; the mineral kingdom the forerunner of the vegetable, and that of the animal, which was impossible without the preceding two. Some vegetables and animals antedate others, but the consumed must exist before the consumer. The simpler primal forms once created, higher forms become possible. The animal kingdom is not derived, exclusively, directly from the vegetable. The lion cannot live on grass like the ox. Proximate principles are only found in cell-formation of the vegetable kingdom ready for the animal. The chemist has never been able to produce these principles. The alchemist looked forward to the time when he could make a man outside of the regular order; the chemist, never. Mineral matter cannot be appropriated by the animal before being elaborated by the vegetable. He was surprised to hear Dr. Spalding say that salt could be taken into the system and appropriated.

*Dr. Spalding*—I did not state it; I raised the question.

*Dr. Patrick*—Salt—the chloride of sodium—goes into the system, penetrates every part of the body, does its work, and comes out of the system, still chloride of sodium. Vegetable life is the larval state of the animal. There is an anatomical reason why the tooth cannot be reconstructed. It cannot repair itself, except in the cementum. The cementum of any animal is precisely like the bone of the same animal; so exactly the same that the anatomist can classify an animal as readily by the cementum as by a bone. In the cementum, as in bone, nature forms a matrix for the deposition of new matter. The tooth hardens at the periphery, and is soft in the centre. The pulp is the remains of the organic matrix of the tooth. Bone, on the contrary, hardens at the centre first. One is centripetal in growth, the other is centrifugal. The tooth being hardened at the periphery cannot reconstruct, except in the cementum. If broken below the cementum it will repair, but not above the pulp. In old age the whole tooth is solid, but the last dentine deposited is not like the first. The dental energies were exhausted.

*Dr. Thompson*, (Topeka, Kan.)—Said that it depends on the powers of protoplasm. The vegetable elaborates the different elements; the animal appropriates the elements after they have been thus elaborated. We can appreciate how it is appropriated by the animal, but we cannot understand how it is elaborated by the vegetable.

Animal structures are but modified protoplasm. Man differs from the lowest orders in having limbs and muscles to control them. He differs in structure, but he is only a larger mass of protoplasm, with rigid materials holding the protoplasm in place. Vegetable protoplasm contains forty elements; animal protoplasm has not been analyzed so exactly. Vegetable protoplasm is almost constant, but animal protoplasm differs greatly. In the teeth it is very dense. The different organs and tissues differ infinitely. The whole question turns upon the ability to appropriate certain elements. With regard to the administration of mineral matter for building up animal tissues, mineral elements are not food, neither are all vegetable elements food. Morphine, for instance, and many other vegetable products are medicinal or poisonous, while much of our so-called food is really medicine.

We cannot direct the elements to any one point with certainty. The organs are selective, and take from the pabulum what they most need. In the administration of food we cannot be certain that it will benefit any one organ. Assimilation is the one great point in nutrition. One organ may be very weak and not able to select the proper elements for its own use, or to derive sustenance from the pabulum.

*Dr. C. W. Spalding*—Said that the gentleman from Illinois had misunderstood him; that the point he had endeavored to make clear was that salt was taken into the system by man and beast, and excreted unchanged, and that he had raised the question whether any part was appropriated, as it had not been determined that all was excreted; that it appeared probable that some part was decomposed and appropriated.

*Dr. E. T. Darby*, (Philadelphia) — Said that he could recall many instances where there was a hunger and craving for inorganic substances, and where the inference from results was that they were appropriated. He had known of a child, with rickets, which had such an inappeasable appetite for lime that it would crawl to the wall and pick the plaster off till it had cleared a space two feet by three. He had suggested the administration of lime in more suitable form. When lacto-phosphate and cod-liver oil had been given, the child stopped eating the plaster. In the same way, when the school-girl eats slate-pencil and chalk, it is because there is a craving for these elements in the system. Thus, when pregnant women have similar cravings, it is not fancy; it is a demand of the system which is satisfied when the proper element is supplied. The continuous administration of these elements for months, and perhaps years, may be necessary to produce results. Until this is done we cannot say that their administration has no effect. It may not always show in one child, or in one generation. He quoted the anecdote of Agassiz, the great naturalist, who advanced the theory of fish-eating for brain development. Meeting on the sea-shore a fisherman's child which had been raised on fish diet, but had a very small head and deficient brain, it was pointed out to him as disproving his theory. He quietly replied: "But what might that boy have been if he had not eaten fish!" And so with the teeth. There is no telling what the result might not be if the lime-salts were administered faithfully and continuously. He believed it would work great results.

*Dr. Atkinson*—Said that he was happy in the fact that the Association had come to its right mind, and begun to investigate principles. The great difficulty lay in calling the same thing by different names, and different things by the same name. What is the function of iron in the system? It is not merely for coloring matter in the blood. The oxide of iron is a constituent of the blood; a slight increase of oxygen gives the sesqui-oxide of iron—the magnet—inviting oxygen to hang about the corpuscles with an oxygenating grip—courting, but not marrying. In this state it is carried throughout the system, and when in the neighborhood of needy territory the greater demand for oxygen in the spent tissues induces a breaking up of the courtship by appropriating the loosely-held oxygen with which the half-equivalent of oxygen making the sesqui-oxide goes, thus demagnetizing the corpuscle, leaving it a non-magnet, to be carried to the lungs and other respiratory tracts, to be again polarized by appropriation of another half-equivalent of oxygen, thus fitting it again to be an oxygen carrier.

Oxygenation and oxidation are the prime movers in nutrient and denutrient activity. Oxygen once within the grasp of the lungs, and not appropriated by the oxygenating process, has its bonds of affinity put to sleep, or made unfit to support respiration. Just ninety-six per cent. is thus rendered unfit for systemic use, and four per cent. is appropriated. This benumbed oxygen has to be vivified or awakened by passing through vegetable tisssue before it is again fitted for animal use.

On motion, the subject was passed.

